# Preparation of Pd-Loaded Hierarchical FAU Membranes and Testing in Acetophenone Hydrogenation

**DOI:** 10.3390/molecules21030394

**Published:** 2016-03-22

**Authors:** Raffaele Molinari, Cristina Lavorato, Teresa F. Mastropietro, Pietro Argurio, Enrico Drioli, Teresa Poerio

**Affiliations:** 1 Department of Environmental and Chemical Engineering, University of Calabria, cubo 44A, Via Pietro BUCCI, 87036 Rende (CS), Italy; cristina.lavorato@libero.it (C.L.); teresafina.mastropietro@gmail.com (T.F.M.); pietro.argurio@unical.it (P.A.); e.drioli@unical.it (E.D.); 2 National Research Council—Institute for Membrane Technology (ITM–CNR) c/o University of Calabria, cubo 17C, Via Pietro BUCCI, 87036 Rende (CS), Italy; t.poerio@itm.cnr.it

**Keywords:** catalysis, ketone hydrogenation, zeolite membranes, secondary porosity, sustainable chemistry

## Abstract

Pd-loaded hierarchical FAU (Pd-FAU) membranes, containing an intrinsic secondary non-zeolitic (meso)porosity, were prepared and tested in the catalytic transfer hydrogenation of acetophenone (AP) to produce phenylethanol (PE), an industrially relevant product. The best operating conditions were preliminarily identified by testing different solvents and organic hydrogen donors in a batch hydrogenation process where micron-sized FAU seeds were employed as catalyst support. Water as solvent and formic acid as hydrogen source resulted to be the best choice in terms of conversion for the catalytic hydrogenation of AP, providing the basis for the design of a green and sustainable process. The best experimental conditions were selected and applied to the Pd-loaded FAU membrane finding enhanced catalytic performance such as a five-fold higher productivity than with the unsupported Pd-FAU crystals (11.0 *vs.* 2.2 mg_product_ g_cat_^−1^·h^−1^). The catalytic performance of the membrane on the alumina support was also tested in a tangential flow system obtaining a productivity higher than that of the batch system (22.0 *vs.* 11.0 mg_product_ g_cat_^−1^·h^−1^).

## 1. Introduction

Reduction processes are among the prevalent transformations in organic chemistry and are widely applied in industrially relevant practices [1]. In the pharmaceutical and biotechnological industries, the synthesis processes for the preparation of a wide range of compounds often imply the conversion of functional groups, the reduction of a carbonyl moiety to the corresponding alcohol being one of the most commonly required intermediary transformations [2,3]. On the industrial scale, however, many well-established and routinely applied technologies for carbonyl reduction need to be revised to meet the more stringent standards of safety and efficiency currently imposed by regulatory agencies in many countries worldwide. Consequently, both industry and academia have placed a special emphasis on the design of more sustainable processes and technologies which can be reliably implemented in large scale production.

Metal-catalyzed homogeneous and heterogeneous reduction of ketones are without doubt reliable routes for the transformation of many organic substrates and are commonly applied in industry [4]. Dihydrogen (H_2_) is usually employed in the process as the ideal reducing agent, owing to its moderate cost and its ability to afford reduction products in high yield and purity [5,6,7]. The drawbacks include the catalyst cost, which can be an issue on a large scale, especially when homogeneous catalysts are used, and the need for specialized equipment for hydrogen storage and safe manipulation. To partially overcome some of these limitations, efforts in the field of catalytic reduction have recently focused on the development of processes which make use of different reductants able to formally transfer an H_2_ molecule to the substrate (*i.e.*, transfer hydrogenation) [8,9,10]. Transfer hydrogenation has thus emerged as an attractive alternative to hydrogenation due to its operational simplicity and the easy availability of cheap hydrogen sources [11,12,13,14,15,16,17]. Furthermore, the possibility of conducting the reduction in water makes this process environmentally friendly [18,19,20]. Despite the exceptional results reported in several lab-scale examples, however, the application of this process in industrial production is still limited.

Catalytic Membrane Reactors (CMRs) represent an emerging technology which promises to achieve energy efficient, clean and easy to scale-up processes [21,22,23,24]. Indeed, CMRs can synergistically combine the advantages of classical metal-catalyzed transfer hydrogenation and those offered by a membrane process [25,26]. The reduction of the substrates and the selective separation of the desired products can be achieved in a single step [27], while the catalyst, immobilized on the membrane surface, can be recovered and reused, thus minimizing the catalyst expenses. Moreover, the reaction can be carried out in continuous or a quasi-continuous manner in a membrane reactor. As a result of the controlled contact time, side-reactions are reduced and the operating life of the catalyst is increased [28]. Nevertheless, despite many successful examples reported in literature, the application of this technology at an industrial level has not been realized yet. A lot of fundamental work is still required in order to demonstrate the feasibility, together with new membrane materials showing better performances.

In this aspect, to the best of our knowledge, the possibility of using hierarchical organized meso/microporous zeolite membranes as potential catalyst support to be used in a CMR has never been investigated. Hierarchically organized microporous zeolites contain a secondary meso- or macroporous network interconnected to the intrinsic microporosity of zeolites and superior external surface area [29,30,31]. The interest in this kind of materials has been rapidly growing since they promise to outperform conventional zeolites in many traditional and emerging applications. In the catalytic field, the presence of porosity at different scales and enhanced surface area improves the performances of hierarchical zeolites with respect to conventional zeolite materials [32,33,34]. Indeed, the incorporation of mesoporosity into zeolites, while keeping unaltered the other features, improves the diffusion rates, the number of active sites of zeolites and their accessibility, resulting in a higher catalytic activity. Moreover, a longer catalyst lifetime and increased selectivity for primary products are also expected, owing to the shortening of the contact time of the catalyzed molecules. While many discrete hierarchical zeolite particles have been tested in suspension for different catalytic process, to the best of our knowledge, there are no reports on the use of zeolite membranes containing an auxiliary porosity.

In this work, a FAU membrane, constituted by nanozeolites assembled on a ceramic substrate, has been prepared and tested as a metal catalyst support. As a result of the assembly of nanozeolites in a thin film, the membrane inherently contains a secondary mesoporous network and increased surface area [35]. The main aim was to demonstrate that FAU nanoparticles can be assembled and fixed onto a (porous) substrate and contain pores at meso-scale level that can be profitably used as metal catalyst support. The catalytic performance, preserving at once the integrity of zeolite layer and its adhesion on the support was also tested in tangential flow mode operation.

Home-made Pd particles as catalyst, different solvents and hydrogen donor sources have been employed. Acetophenone (AP) has been chosen as model substrate for aromatic ketone hydrogenation. Commercial FAU zeolite micro-seeds and Pd-loaded FAU membranes precursor were also compared.

## 2. Results and Discussion

### 2.1. Preliminary Remarks

Hydrogenation of acetophenone (AP) is a widely studied process. Its corresponding alcohol, namely 1-phenylelthyl alcohol or phenyl ethanol (PE), is a common precursor for the preparation of analgesic and anti-inflammatory drugs, as well as fragrances and perfumes [27,36,37]. The selective conversion to PE is one of the major challenges encountered in the hydrogenation of ketones like AP, since competitive hydrogenation of the aromatic ring or hydrogenolysis of the corresponding alcohols can also occur [38,39]. AP hydrogenation has been studied with various heterogeneous catalysts using molecular hydrogen, sometimes with very good results in term of selectivity towards PE [40,41,42]. On the contrary, transfer hydrogenation of AP using different hydrogen donors has been typically accomplished by using transition metal complexes as homogeneous catalysts [43,44,45].

Here, we evaluate the catalytic properties of a Pd-loaded FAU membrane constituted by hierarchically assembled nanocrystals in the heterogeneous transfer hydrogenation of AP. This membrane inherently contains an auxiliary mesoporous structure and an enhanced external surface area which could be profitable for enhancing the number of active sites as well as their accessibility, thus shortening the substrate-catalyst contact time. A fine balance between reactivity and selectivity is expected which would finally result into a higher catalytic activity and an increased selectivity of the products.

### 2.2. Pd-FAU Crystals: Effect of Pd Content and Exchange Temperature

Synthesis of Pd-loaded FAU crystals resulted in a gray powder material. The Pd-FAU crystals were prepared from the corresponding Na-FAU zeolite by ion exchange by using a PdCl_2_ aqueous solution as palladium precursor.

**Figure 1:**
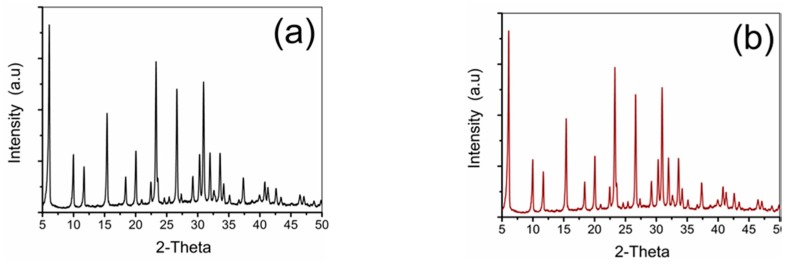
XRD patterns collected on pristine FAU seeds (**a**) and Pd-FAU(0.5-15) sample (**b**).

XRD patterns on the three samples: Pd-FAU(0.5-15), Pd-FAU(0.5-60) and Pd-FAU(1-60) were collected and compared with the powder pattern of the pristine FAU seeds (Figure 1a) with similar results. As shown in Figure 1b for Pd-FAU(0.5-15), the sample exhibits typical diffraction peaks that are characteristic of a pure FAU phase, confirming that the crystalline structure is kept unaltered after the exchange and the subsequent reduction of Pd^2+^.

Moreover, for all the samples, no Pd nanocrystal diffraction peaks are observed in the XRD patterns. These results can be attributed to the presence of typical FAU peaks located at the 2-Theta values of interest, so that the eventual presence of weak diffractive peaks from Pd nanoparticles is hidden. On the other hand, it can be also a sign of the good dispersity of active Pd nanoparticles on the surface of the zeolite supports. Unfortunately, these outcomes prevented us from performing any further analysis to determine, for example, a reliable Pd mean particle size.

**Figure 2:**
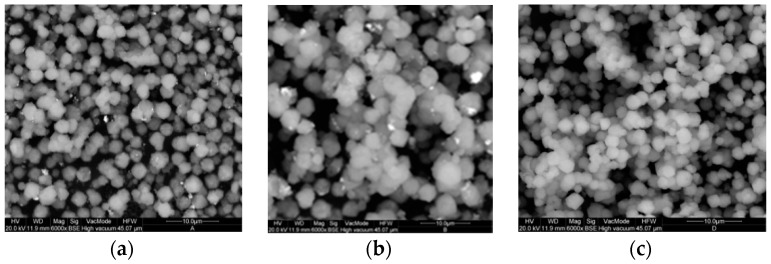
SEM images in BSE mode of Pd-FAU(0.5-15) (**a**); Pd-FAU(0.5-60) (**b**) and Pd-FAU(1-60) (**c**) samples.

**Figure 3:**
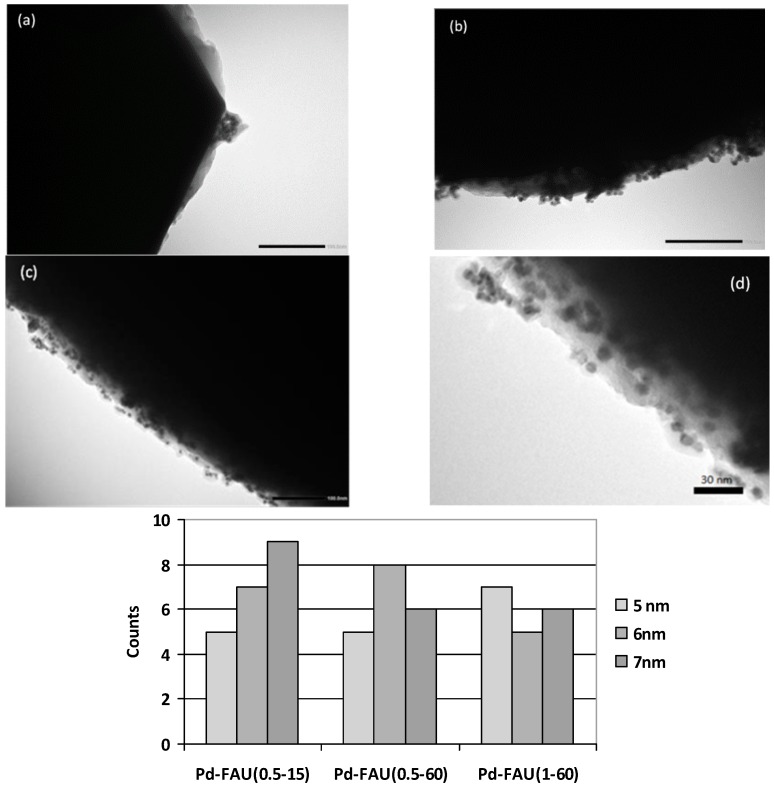
TEM images of Pd-FAU(0.5-15) (**a**); Pd-FAU(0.5-60) (**b**,**d**) and Pd-FAU(1-60) (**c**) samples and the pertinent histograms showing the Pd particle size distributions. Scale bar: (**a**–**c**) 100 nm; (**d**) 30 nm.

The texture of the samples was analyzed by SEM, using the Back Scattering Electrons (BSE) method to detect contrast between areas with different chemical compositions, while EDX analysis were performed in order to establish the effective Pd content. From Figure 2a,b it is possible to observe visible shiny points, which are self-standing Pd particles quite big (the bar scale is 10 µm) with a heterogeneous distribution in the particle size (from *ca.* 500 nm up to 1 µm). A “suffused shiny appearance” is also visible on the crystal surface, from which, as it can be seen from the TEM images of Figure 3 described below, it can be supposed that Pd is quite homogeneously distributed on the FAU crystal surface (at a microscale level). The mean Pd content determined with EDX by mapping different areas of the samples was 1.0 and 1.5 wt % for Pd-FAU(0.5-15) and Pd-FAU(0.5-60), respectively, proving a positive effect of temperature on the efficacy of the exchange process.

On the contrary, a careful inspection of the Pd-FAU(1-60) sample revealed the absence of any discrete self-standing Pd particles. Surprisingly, it seems that Pd species are quite uniformly distributed on the zeolite surface. The presence of palladium was confirmed by EDX analysis. The measured Pd content was 4.2 wt %, indicating a higher degree of cation exchange within the zeolite framework. The morphology and size of the Pd nanoparticles loaded on the zeolite microcrystals were investigated by Transmission Electron Microscopy (TEM) in order to get information at a nanoscale level. The transmission electron microscopy images of Pd-FAU(0.5-15) (a), Pd-FAU(0.5-60) (b) and Pd-FAU(1-60) (c) samples are shown in Figure 3, along with the pertinent histograms showing the particle size distributions (d).

Both Pd-FAU(0.5-60) (Figure 3b) and Pd-FAU(1-60) (Figure 3c) samples show the presence of distinct Pd nanoparticles homogeneously dispersed on the external surface of zeolite crystals. The Pd nanoparticles feature a quasi-spherical shape and quite uniform particle size, with values ranging from 5 to 7 nm for all the samples. However, in Pd-FAU(0.5-60), some nanoparticles also form agglomerates of greater dimensions (30–50 nm) which stably adhere to the external surface of the zeolite crystals. On the contrary, in Pd-FAU(0.5-15) Pd particles are not uniformly and densely distributed on the zeolite surface (Figure 3a). The surface area of the zeolite is only partially covered by Pd nanoparticles, which are basically present as agglomerates (100–150 nm) of particles of smaller dimensions.

The textural properties of the microcrystalline Pd-FAU samples were investigated by determining the porous volume and surface area of the host material from nitrogen adsorption isotherms, compared with data obtained for pristine FAU microcrystals (Table 1).

As it can be seen, the Pd-FAU(1-60) shows a lower BET surface area and micropore volume if compared with samples Pd-FAU(0.5-15) and Pd-FAU(0.5-60), which can be attributed to the formation of Pd nanoclusters within the micropores of the zeolite as well as on the external surface. The nanoparticles loaded on the external surface are mostly exposed and consequently readily accessible while the Pd species included into the pores are not easily accessible to the reacting molecules and scarcely contribute to the catalytic activity. The results of the catalytic tests carried out with the three samples under the same experimental conditions are reported in Table 2.

The degree of conversion and the selectivity values for Pd-FAU(0.5-60) are higher than Pd-FAU(0.5-15), as expected considering the higher amount of Pd catalyst loaded on the zeolite seeds. Furthermore, concerning the Pd-FAU(0.5-15) sample, the scarcity of Pd particles present on the external zeolite surface lowers the active surface area of the catalyst and, accordingly, its catalytic efficiency. On the contrary, and quite surprisingly, a lower conversion is observed for Pd-FAU(1-60). This behavior can be explained by taking into account the distribution of the active Pd metal sites deep inside the zeolite framework, which cannot be easily reached by the reacting molecules.

On the basis of the experimental results, it can reasonably hypothesized that the Pd particles on the surface of the zeolite crystals exhibit the best catalytic efficiency compared to the Pd sites confined inside the zeolite pores. The Pd-FAU(0.5-60) sample was selected for further testing of the catalytic process.

### 2.3. Influence of the Solvent and the Hydrogen Donor

Although the role of the catalyst is of primary importance to achieve both high activity and selectivity, the solvent selection is of great relevance, as well. Indeed, it has been demonstrated that the solvent can sensibly affect the catalyst activity and selectivity [46] as a result of different solvent/reactant and solvent/catalyst interactions. In this work, we tested different solvents as well as different hydrogen donors. The results obtained in terms of selectivity, conversion and yield are reported in Table 3.

As it can be seen, the catalytic activity (conversion) varies according to the following pattern: HCOONa/water > ethanol > 2-propanol and it seems to follow the decreasing polarity of the solvents as well as the decreasing dehydrogenation activity of the hydrogen donor [47,48]. Indeed, formic acid and its salts are strong hydrogen donors [49]. An influence of the solvent nature on selectivity and yield was also observed. In particular, both increase with decreasing alcohol polarity, while a maximum is observed for the HCOONa/water system.

**Figure 4:**
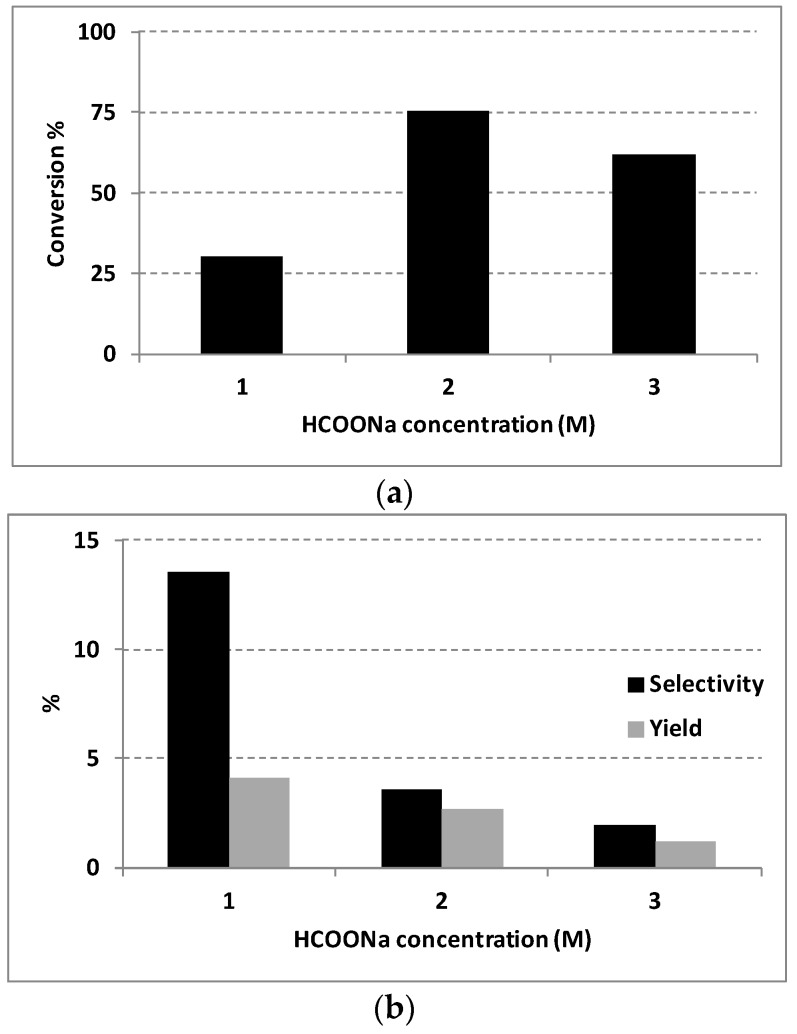
Acetophenone conversion (**a**); selectivity to PE and PE yield (**b**) for different HCOONa concentration (Operating conditions: acetophenone = 0.026 M, Pd-FAU(0.5-60) = 2.5 g·L^−1^, 60 °C, 50 mL water, 24 h).

Since the HCOONa/water system seems to guarantee better results for the transfer hydrogenation process, we also investigated the effect of HCOONa concentration on the AP conversion. Results are summarized in Figure 4.

A higher selectivity to PE was observed using HCOONa 1 M as hydrogen source, even if a lower catalytic activity is also observed, while the increase of the concentration up to 3 M significantly decreases the selectivity value.

Since formate features both a Brønsted and Lewis basicity (being an efficient hydrogen donor), we can speculate that it can also act as acidity inhibitor, especially when high concentrations are used. Indeed, the resultant pH value of the reaction media (starting pH is *ca.* 10) also increase by increasing the formate concentration. Moreover, the interaction of formate ions with the zeolite framework can lead to a change in the electron distribution or charge on the support, with a consequent variation of the metal/support interactions, and ultimately, of the catalyst activity. Finally, the poisoning or leaching of the Pd catalyst can also occur, caused by the increased concentration of formate molecules.

For comparative purposes, catalytic experiments were conducted in the best selected condition using pristine FAU crystals and home-made Pd particles, prepared following the procedure described for the preparation of Pd-loaded FAU crystals. Table 4 summarizes the results. As it can be seen, even if the Pd-FAU(0.5-60) sample shows a lower catalytic activity with respect to both the unsupported Pd particles and the pristine FAU crystals, it shows a higher selectivity, yield and productivity if compared to both of them. The best performance of Pd-FAU(0.5-60) can be most likely attributed to the synergy resulting from (i) Pd particles well dispersed on the larger surface area of the zeolite support when compared to the free Pd catalyst and (ii) a selective effect of the porous zeolite framework.

### 2.4. Pd-FAU Membrane: Synthesis, Characterization and Catalytic Activity

**Figure 5:**
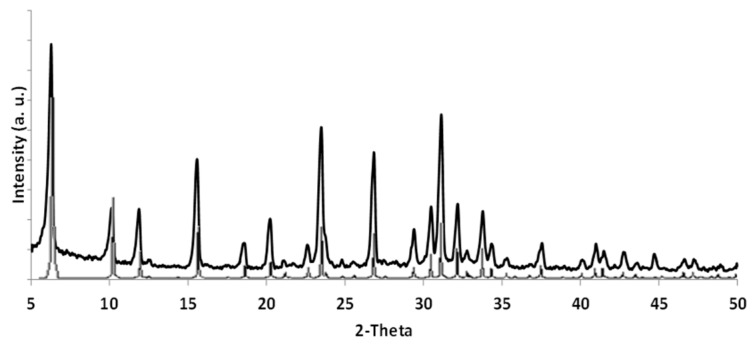
XRD pattern of the FAU nanocrystalline layer synthesized after 6 h of hydrothermal treatment.

The membrane synthesis was carried out according to the procedure described in Section 3.2 obtaining a thin and dense zeolite top layer on the support surface. XRD analysis, performed on a sample scratched from the support surface, indicated the formation of a well-crystallized, pure FAU phase (Figure 5).

**Figure 6:**
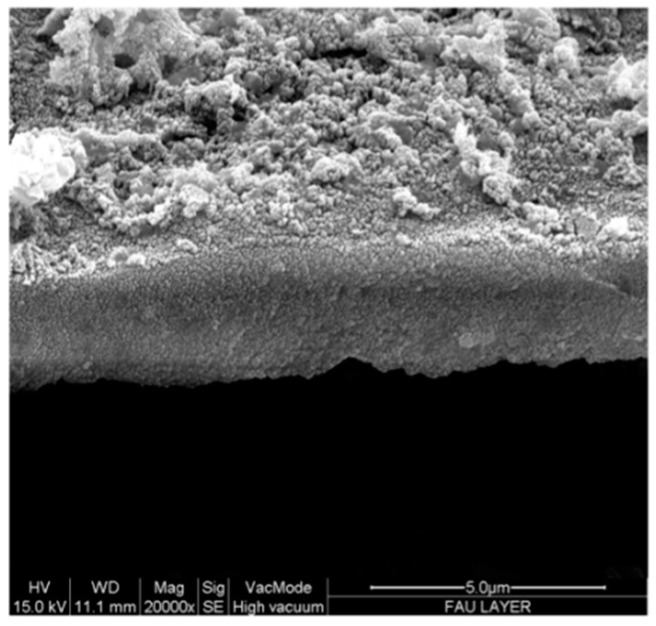
SEM side view of a self-standing fragment of the FAU nanocrystalline layer split from the support surface obtained after 6 h of hydrothermal treatment.

SEM images collected on the sample showed that the FAU layer has a thickness of *ca.* 2 μm and presents a granule-like texture. A careful inspection revealed that the layer was constituted by closely packed intergrown nanocrystals, whose dimension was *ca*. 20–30 nm (Figure 6). EDX analysis showed that the zeolite layer had a Si/Al ratio of *ca.* 2.5.

**Figure 7:**
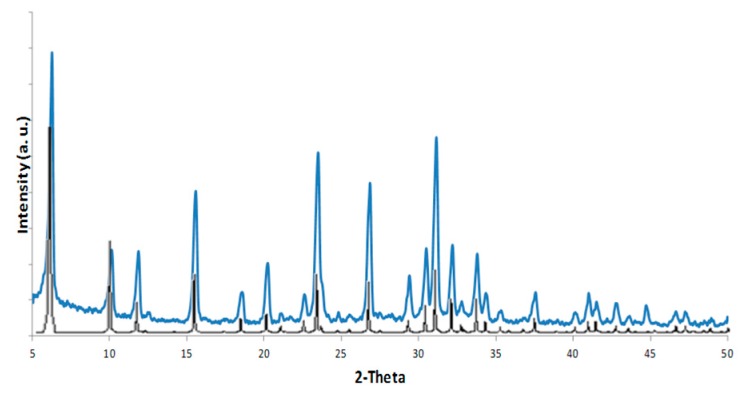
XRD powder pattern collected on a Pd-loaded zeolite.

The membrane was exchanged by using the procedure described in Section 3.3 for the FAU(0.5-60) sample, with a final Pd content of *ca*. 1.4% determined by EDX. XRD analysis performed on a Pd-loaded sample indicated that crystallinity and phase purity of the zeolite layer were fully preserved upon treatment (Figure 7).

The Pd-FAU membrane was tested under the conditions described in Section 3.5. Typically, a lowering of the catalytic activity should be expected for the supported Pd-FAU membrane with respect to the bare Pd-FAU crystals as a result of the decrease of the active surface area, as well as more pronounced mass transport limitation effects. Indeed, even if the membrane is constituted by nanozeolites, they are not isolated domains. Because of intraparticle growth, zeolite crystals coalesce, forming a unique extended macro-structure. As a consequence, it can be seen from Table 5 that the values of PE productivity (based on the total amount of catalyst/support and on the sole amount of loaded Pd) obtained with the Pd-FAU membrane are much higher than Pd-FAU(0.5-60). 

**Figure 8:**
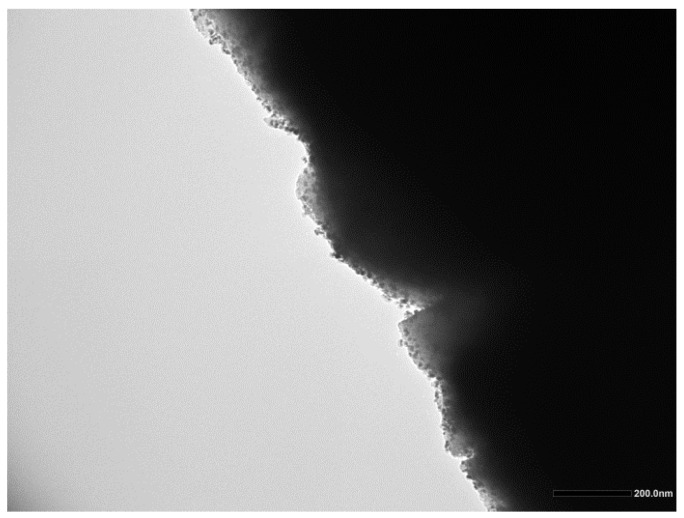
TEM image of the Pd-FAU membrane.

On the basis of the obtained results and taking into account the microstructure of the zeolite membrane support, we can speculate that the nanozeolite based membrane is able to guarantee a higher surface area compared to that of the pristine FAU seeds [50]. This surface area is thus available for palladium deposition, allowing a better metal dispersion on the zeolite membrane surface. Furthermore, the presence of a secondary mesoporosity, which can originate among crystallite domains, can also minimize the mass transport resistance, facilitating the diffusion of the AP molecules to the catalytically active zeolite wall sites. To confirm this hypothesis, TEM (Figure 8) and BET (Table 6) analyses were performed on the Pd-FAU membrane.

The image of Pd-FAU membrane shows the presence of well dispersed Pd nanoparticles on the external surface of the zeolite layer, with no significant variation in their particle size and shape with respect to the Pd-FAU microcrystalline samples. We did not observe the presence of any agglomerate in the samples investigated.

BET analysis (see Table 6) indicates a high external surface area which is about 50% of the total surface area for the Pd-FAU membrane, most likely due to the presence of nanozeolites constituting the membrane layer. Moreover, the value of the median pore width of *ca*. 5 nm confirms the formation of mesopores. The diffusion contribution in the zeolite pore (average pore width 1.9 nm) will be negligible compared to the diffusion in the inter-crystalline mesoporosity originated within the membrane layer. It must be observed that extra-zeolite mesoporosity is generated only in the membrane layer. Indeed, DLS measurements [51], carried out on synthesized FAU nanocrystals, indicated mean particle size distribution values in the range of 50–59 nm. Moreover, the high pH value (*ca*. 10) used in the studied catalytic reaction favors the formation of stable nanocrystals suspension, thus excluding any aggregates formation. In other words, mesoporosity will dominate the reagent diffusion to the catalytically active zeolite wall sites. This finding confirms the results recently obtained on the use of this hierarchical membrane for gas de-watering [52].

Zeolite layers having low diffusional resistance (owing to the presence of micro-, meso- and macroporores) allow substantial improvements of selectivity when the target product is the intermediate of a successive reaction pathway or when other competitive reactions occur, since they are able to provide reduced reactant/catalyst contact-time. Furthermore, the high external surface area guaranteed by nanozeolites also provide a suitable surface for the homogeneous dispersion of the Pd catalyst (the post synthesis modification of FAU nanozeolites results into the dispersion of Pd particles mainly on the external surface of the zeolite), which in turn implies higher productivity value.

To prove the reproducibility of the results and the reliability of this catalytic system upon time, the Pd-FAU membrane was reused consecutively for three times. The results are reported in Table 7. As it can be seen, despite a significant lowering of the conversion observed after the first cycle, the system is quite stable in terms of yield and productivity over time. Selectivity towards the formation of PE is low, but it is also preserved. These results indicate a stability of the membrane but its catalytic function must be further investigated before considering possible future industrial applications of membrane reactors manufactured with hierarchical porous membranes combining the properties of suitable zeolites and catalysts.

**Scheme 1:**
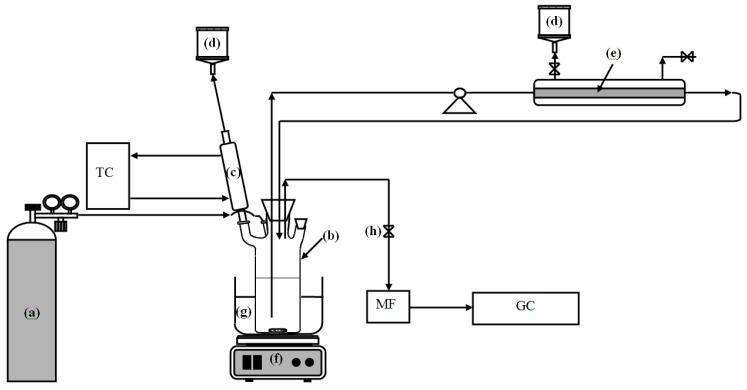
Schematization of the tangential-flow membrane system: (**a**) argon cylinder; (**b**) vessel; (**c**) bubble chiller; (**d**) degassing system; (**e**) tubular membrane; (f) magnetic stirrer with hot plate; (**g**) oil bath; (**h**) sampling valve; (MF) microfiltration of samples; (GC) gas chromatograph; (TC) temperature controller.

The Pd-FAU membrane was also tested in tangential-flow mode to check its adhesion on the support. To do this test the membrane was housed in the system described in Scheme 1. The results are reported in Table 8 and compared with that obtained in the batch system.

It can be seen that when the transfer hydrogenation reaction takes place in tangential-flow mode an increase of yield, conversion, selectivity and productivity is observed compared to the batch. This improved performance of the membrane, when operated in tangential flow mode, can be ascribed to predominance of convection in the mesoporosity over the diffusion which happens in the batch system. These results further confirm the efficacy of the proposed catalytic system for the studied reaction.

However, it is important to underline that various operating parameters (e.g., contact time of reacting solution with the catalytic membrane, pump flow rate, type of catalyst, *etc.*) need to be investigated and optimized in a suitable membrane reactor.

Concerning the Pd catalyst is must be observed that it was only a first choice to test the catalytic function. In Table 9, the conversion (C), selectivity (S) and yield (Y) obtained with the Pd-FAU membrane are compared with the results obtained with another Pd catalyst [53], supported on silica particles, used in the heterogeneous transfer hydrogenation of AP. Comparable yield (%) of PE has been obtained even if a higher ratio Pd/[AP] and higher temperature was used in ref. [53].

However, by looking at other examples reported in literature, [54,55,56] it is clear that the catalyst performance is strongly improved by the choice of catalytic system including the nature of the metal, the degree of crystallinity as well as the type of metal catalyst support. The results obtained with our catalytic system, where the micro/mesoporous zeolite membrane was used as catalyst support, could be further improved by selecting a different metal catalyst among those which appear to be more active in the transfer hydrogenation of AP.

## 3. Materials and Methods

### 3.1. Materials

Porous α-Al_2_O_3_ asymmetric tubes (I.D. = 7 mm, O.D. = 10 mm, length = 100 mm, d_pore_ = 200 nm, IKTS, Hermsdorf, Germany) were used as supports for zeolite membrane growth. The supports were rinsed in acetone in an ultrasonic cleaner for 1 h, then boiled in distilled water for 1 hour and dried in a furnace at 100 °C for 2 h to remove any dusty and oily materials. NaX zeolite crystals (Sigma-Aldrich, Milan, Italy) having a mean crystal size of about 2 μm and a Si/Al ratio of 1.5 were used for seeding the inner supports surface and free standing supports in the preliminary catalytic tests. All the reagents used for preparing the synthesis solution were purchased from commercial sources and used without further purification: sodium aluminate (53%–55% Al_2_O_3_, Carlo Erba Reagenti, Milan, Italy), sodium silicate solution (NaOH 14 wt % and SiO_2_ 27 wt %, Sigma-Aldrich), and sodium hydroxide (NaOH pellets, 97%, Carlo Erba Reagenti).

Acetophenone Reagent Plus (C_8_H_8_O, 99% purity), from Sigma Aldrich, was used as substrate. Palladium(II) chloride (PdCl_2_, 99% purity) and sodium borohydride (NaBH_4_, 99% purity) from Sigma Aldrich, were used to synthesize home-made Pd-FAU and Pd particles. Silver nitrate (AgNO_3_, purity 99.0%) was used to detect the presence of chloride ions in solution after synthesis. Sodium formate (HCOONa, 99+% purity) from Sigma Aldrich, isopropanol (C_3_H_8_O, 99.7% purity), absolute ethanol (C_2_H_6_O, 99.8% purity) from Carlo Erba Reagenti were used during the catalytic tests as both solvents and reducing agents. Sodium hydroxide pellets (NaOH, 97% purity) from Sigma Aldrich and hydrochloric acid (HCl, 37% *w*/*w*) from Carlo Erba Reagenti were used to adjust the pH. Ultrapure water was obtained from Milli-Q equipment by Millipore (Billerica, MA, USA).

### 3.2. Preparation of the Nanozeolite-Based Membrane

The detailed procedure for the preparation of the nanozeolite-based membrane has been described elsewhere [35]. The supports were boiled in a NaOH solution 0.1 M for 1 h in order to create and activate the unsaturated hydroxyl groups on the surface. The seeding procedure was carried out by carefully coating a seed suspension on the inner support surface to achieve a uniform deposition on the whole surface area. The amount of seeds (*wt*/*wt* %) layered onto the inner support surface was *ca*. 2% with respect to the starting amount of SiO_2_ used in the successive synthesis of the zeolite nanocrystals.

The seeded support was then placed into a Teflon-lined autoclave and a thermal treatment was performed in a pre-heated furnace at 160 °C for 6 h without any solvent, to promote the condensation reaction between the hydroxyl functions on the seeds and those on the support.

FAU zeolite membranes were then prepared by secondary growth method. A synthesis solution with the following starting molar composition: 4.3SiO_2_:1Al_2_O_3_:15.2Na_2_O:321.4H_2_O [35] was used for the room temperature synthesis. The reaction gel system was prepared at ambient temperature, by gently adding a clear water solution containing the sodium aluminate and the sodium hydroxide reagents to the sodium silicate solution in a LDPE bottle under continuous stirring. A clear suspension was obtained, which turned into a dense milky gel in few minutes. The synthesis solution was then poured in a cylindrical vial containing the seeded support (wrapped with Teflon tape in order to avoid crystallization on the outer surface). The vial was horizontally placed in a conventional oven at 30 °C for 24 h. After this treatment the support was transferred in a Teflon-lined autoclave for thermal cure with a synthesis solution having the following molar composition 13.5SiO_2_:1.0Al_2_O_3_:15.8Na_2_O:509H_2_O [35]. The autoclave was horizontally placed in the pre-heated furnace for the hydrothermal treatment at 80 °C for 6 h. The obtained membranes were then accurately washed with distilled water and dried at 80 °C overnight before characterization with XRD, SEM, EDX, TEM and BET.

### 3.3. Preparation of Pd-Loaded FAU Crystals and Membrane

The Pd-FAU crystals were prepared from the corresponding Na-FAU zeolite (1 g) by ion exchange by using a PdCl_2_ aqueous solution containing 0.5 mg/mL of the palladium salt and 1 g of FAU seeds. The suspension was continuously stirred for 72 h at 15 °C or 60 °C during the cation exchange. Another Pd-FAU sample was prepared by using 1 mg/mL of the palladium salt and a temperature of 60 °C in order to enhance the degree of cation exchange. After the exchange step, the Pd-FAU crystals were filtered and washed. Afterword the Pd-FAU crystals were treated with a NaBH_4_ solution to reduce the Pd^2+^ ion exchanged within the zeolite structure, washed and dried. The obtained samples were labeled as Pd-FAU(x-y), where x and y indicate the initial PdCl_2_ amount and temperature used in the cation exchange, respectively. Then, the three prepared samples are named Pd-FAU(0.5-15), Pd-FAU(0.5-60) and Pd-FAU(1-60).

The Pd-loaded membrane was prepared following the same procedure using a PdCl_2_ aqueous solution containing 0.5 mg/mL of the palladium salt, which was continuously stirred for 72 h at 60 °C. The membrane was wrapped with a Teflon tape to avoid the deposition of palladium particles on the outer surface of the support. The membrane was carefully washed before and after the treatment with a NaBH_4_ solution for reducing the exchanged palladium ion, and then it was dried.

The catalytic supported membrane and all catalysts, before to be dried, were accurately washed with Milli-Q water in order to remove chloride ion until chlorine was not detected in the washing solution using the argentometric method. This method was used because small quantities of chloride ions can alter the properties of the catalyst because they can have an activating or poisoning effect. These alterations in the activity of the catalyst are often caused by simple physical blocking of some adsorption sites [27].

### 3.4. Characterization

The morphology of the pristine FAU seeds, the Pd-loaded FAU seeds and the Pd-loaded nanozeolite membrane were investigated by using a LEO 400 scanning electron microscope (SEM, Cambridge Zeiss, Cambridge, UK) and a JEM 1400 Plus electron microscope at 100 kV (TEM, Jeol, Akishima, Tokyo, Japan). The Si/Al ratio and the Pd content were determined by energy dispersive X-ray (EDX) performed with a Phoenix instrument (SUTW Detector, analyzer: Si/Li crystal, EDAX, Naperville, IL, USA). The crystal phase of the zeolite crystals and membranes was identified before and after the Pd(II) exchange by X-ray powder diffraction (XRD) performed with a PW 1730/10 X-ray diffractometer (Ni filtered Cu Ka_1_ + Ka_2_, λ = 1.542 Å, Philips, Amsterdam, The Netherlands).

The textural characterization of microcrystalline FAU, Pd-FAU(0.5-15), Pd-FAU(0.5-60), Pd-FAU(1-60) and Pd-loaded membrane was carried out by means of absorption of N_2_ at 77 K with a Tristar II analyzer (Micromeritics, Norcross, GA, USA). A self-standing fragment of the zeolite membrane, obtained by a careful exfoliation of the original supported membrane, was used for BET measurement. The sample tube was filled with a known amount of catalyst or catalytic membrane and degassed *in situ* by heating at 300 °C and evacuated for at the least 8 h, in order to remove any adsorbed impurities prior to the adsorption measurements. The volume of nitrogen adsorbed onto the sample at the temperature of liquid nitrogen (−190 °C) was measured as a function of the relative pressure. The apparent surface area values were calculated from nitrogen adsorption isotherms by using the BET equation. Total micropore area and volume were calculated by applying the t-plot method. Mesopore surface area and volume were estimated from the BJH adsorption cumulative surface area and volume taking into account the volume adsorbed between the partial pressures 0.2 and 0.9 in the absorption branch. External surface area was calculated by applying the t-plot method. The pore size was estimated from the adsorption average pore width (4 V/A by BET) and BJH adsorption average pore diameter (4 V/A).

### 3.5. Activity Tests and Product Analysis

#### 3.5.1. Procedure of Catalytic Tests Using Pd-FAU, Pd, and FAU Catalysts

The catalytic tests were carried out in a 50 mL batch reactor, equipped with a condenser working with inlet water at 10 °C, and three outputs in its top section for sampling, argon blowing and degassing. Before starting all catalytic tests the system was purged with argon for 30 min for O_2_ removal. The batch was magnetically stirred during the catalytic tests. 2-propanol or ethanol were used as both solvents and hydrogen donors, while sodium formate was used as hydrogen source with water as solvent. To avoid solubility problem, AP concentration was fixed well below its corresponding saturation concentration in water. The reaction conditions were as follows: 0.1512 mL of AP corresponding to 1.29 µmol and to an instantaneous initial concentration of 0.026 M in the reaction batch; 2.5 g·L^−1^ of particulate catalysts in the case of: FAU seeds, Pd powder and Pd-FAU seeds, or 0.0125 g *ca.* of the supported Pd-FAU membrane; sodium formate 1, 2 or 3 M; stirring rate of 500 rpm to exclude diffusion effects; reaction temperature of 60 °C.

#### 3.5.2. Procedure of Catalytic Tests Using Pd-FAU Membrane

The catalytic tests, conducted using the Pd-loaded nanozeolite supported membrane, were carried out in 1 L batch reactor, similar to that previously described. The solution volume used for these tests was 300 mL which completely covered the tubular membrane. The catalytic tests achieved with membrane were carried out using the following operating conditions, obtained in the batch tests: acetophenone = 0.026 M, catalyst = 0.0125 g, T = 60 °C, solvent: 300 mL water, HCOONa 1 M.

Samples were recovered and analyzed after 24 h of reaction. The samples were filtered through a GH-Polypro membrane to remove the catalyst before the analysis. In the catalytic tests in aqueous media, all withdrawn samples were diluted with 50% of ethanol before to be filtered in order to avoid adsorption of reactant and products on the polypropylene membrane. Detection of possible products was performed by a model 6890 N gas chromatograph (Agilent, Santa Clara, CA, USA) equipped with a FID detector and a Supelcowax™ 10 capillary column (fused silica, 10 m × 0.1 mm × 0.1 μm film thickness). All the experimental tests were repeated three times to ensure the consistency of the results and the reproducibility. The Pd-FAU membrane was also tested in a tangential-flow system shown in Scheme 1.

The obtained results were elaborated as follows:

(1) $$\text{Acetophenone conversion }=\frac{\text{reacted moles of acetophenone}}{\text{initial moles of acetophenone}}\times 100$$

(2) $$\text{Selectivity to phenylethanol }=\frac{\text{produced moles of phenylethanol}}{\text{reacted moles of acetophenone}}\times 100$$

(3) $$\text{Phenylethanol yield }=\frac{\text{produced moles of phenylethanol}}{\text{initial moles of acetophenone}}\times 100$$

(4) $$\text{Phenylethanol productivity }=\frac{\text{produced mg of phenylethanol}}{\text{g of catalyst}\times \text{h}}\times 100$$

## 4. Conclusions

It was possible to prepare a FAU membrane constituted by nanozeolites assembled on a ceramic substrate and containing a catalytic function obtained by Pd loading. The catalytic membranes performed the catalytic hydrogenation of acetophenone (AP) to phenylethanol (PE) using water as solvent, sodium formate as hydrogen and electron donor. This is an interesting perspective for carrying out chemical transformations of organic substrates using green chemistry methods.

The results of the preliminary tests in batch by using FAU crystals as catalyst support to investigate the influence of some operating conditions on the system performances, indicated best results in terms of PE yield by finely modulating the Pd content and the HCOONa concentration.

The Pd-loaded FAU membrane, operated as determined in the preliminary tests, was employed for the reduction of AP obtaining a productivity five-fold higher than the unsupported Pd-FAU crystals.

The results obtained for Pd-loaded FAU crystals and membrane clearly indicate that hierarchical porous membranes are a promising material which can be effectively used as catalysts support for the selective reduction of AP. Some advantages of using immobilized hierarchical assembled nanostructures over the powder ones consists in avoiding the separation of the catalyst from the reacting media and a better control of the contact time substrate-catalyst thanks to the convection through the secondary mesoporous network of the membrane. 

Further research work will be required to find a better catalyst and optimize the operating system parameters of the membrane reactor to improve the process performance. Moreover, the modulation of the multimodal microstructure of the zeolite membrane, especially on fine control of pore size distribution, would be a highly desired attribute for producing high performance catalytic systems.

## Figures and Tables

**Table 1:** BET measurements of pristine and Pd-loaded FAU-microcrystals

	Microcrystalline FAU	Pd-FAU(0.5-15)	Pd-FAU(0.5-60)	Pd-FAU(1-60)
BET Surface Area (m^2^/g)	616	751	727	267
t-plot External Surface Area (m^2^/g)	51	73	67	127
t-plot Micropore Surface Area (m^2^/g)	566	678	660	140
t-plot micropore volume (cm^3^/g)	0.23	0.27	0.26	0.06
Adsorption average pore width (4V/A by BET) (nm)	1.7	1.8	1.9	2.5

**Table 2:** Acetophenone conversion, selectivity to PE and PE yield with the three catalyst samples.

Catalyst	Conversion %	Selectivity %	Yield %
Pd-FAU(0.5-15)	13.08	0.52	0.07
Pd-FAU(0.5-60)	15.27	2.60	0.40
Pd-FAU(1-60)	10.36	1.25	0.13

Operating conditions: acetophenone = 0.026 M, Pd-FAU = 2.5 g·L^−1^, T = 60 °C, 50 mL of 2-propanol, 24 h.

**Table 3:** Acetophenone conversion, selectivity to PE and PE yield with different hydrogen donors and solvents.

Hydrogen Donor	Solvent	Conversion %	Selectivity %	Yield %
HCOONa 2M	Water	75.43	3.57	2.69
2-propanol	2-propanol	15.27	2.60	0.40
Ethanol	Ethanol	32.05	0.35	0.11

Operating conditions: acetophenone = 0.026 M, Pd-FAU(0.5-60) = 2.5 g·L^−1^, 60 °C, 24 h.

**Table 4:** Acetophenone conversion, selectivity to PE and PE yield for Pd-FAU(0.5-60), Pd and FAU.

Catalyst	Catalyst Amount	Conversion %	Selectivity %	Yield %	Productivity based on Pd content (mgPE g_Pd_^−1^·h^−1^)
Pd-FAU(0.5-60)	0.125 g Pd content 1.5% (1.88 mg)	30.37	13.56	4.12	144.6
Pd	3.75 mg	40.40	8.00	3.23	56.8
FAU	0.125 g	51.41	0.26	0.13	/

Operating conditions: acetophenone = 0.026 M, 60 °C, water 50 mL, HCOONa 1 M, 24 h.

**Table 5:** Comparison of the PE productivity by using Pd-FAU(0.5-60) and Pd-FAU supported membrane.

Catalyst (*)	Productivity (mg g_cat_^−1^·h^−1^)	Productivity Based on Pd Content (mgPE g_Pd_^−1^·h^−1^)
0.125 g of Pd-FAU(0.5-60)	2.2	144.6
0.0125 g of Pd-FAU on membrane	11.0	731.3

(*) 0.0125 g of Pd-FAU(0.5-60) catalyst gave no detectable values.

**Table 6:** BET measurements of Pd-FAU(0.5-60) and Pd-FAU supported membrane.

	Pd-FAU(0.5-60)	Pd-FAU Membrane
BET Surface Area (m^2^/g)	727	473
t-plot External Surface Area (m^2^/g)	67	229
t-plot Micropore Surface Area (m^2^/g)	660	243
BJH Adsorption cumulative surface area of mesopores (m^2^/g)	-	219
t-plot micropore volume (cm^3^/g)	0.26	0.19
BJH Adsorption cumulative volume of mesopores (cm^3^/g)	-	0.43
Adsorption average pore width (4 V/A by BET) (nm)	1.9	4.8
BJH Adsorption average pore diameter (4 V/A) (nm)	-	7.9

**Table 7:** Comparison of yield, conversion, selectivity to PE and productivity obtained by re-using the Pd-FAU membrane in batch for 3 times.

Pd-FAU Supported Membrane	Yield %	Conversion %	Selectivity %	Productivity (mgPE g_cat_^−1^·h^−1^)
Run 1	0.35	62.06	0.56	11.0
Run 2	0.35	30.99	1.11	10.8
Run 3	0.27	35.54	0.77	8.6

Operating conditions: acetophenone = 0.026 M, catalyst = 0.0125 g, T = 60 °C, solvent: 300 mL of water, HCOONa 1 M, time course of each run 24 h.

**Table 8:** Comparison of yield, conversion, selectivity and productivity obtained by using the Pd-FAU membrane in batch and housed in a tangential-flow system.

Pd-FAU Membrane	Yield (%)	Conversion (%)	Selectivity (%)	Productivity (mgPE g_cat_^−1^·h^−1^)
Batch reactor	0.35	62.06	0.56	11.0
Tangential-flow reactor	0.69	93.90	0.75	22.0

**Table 9:** Conversion (C), selectivity (S) and yield (Y) of the Pd-FAU membrane compared with a similar Pd catalytic system.

Catalyst	Catalyst Amount [g]	AP [M]	HD ^a^ [M]	T [°C]	C [%]	S [%]	Y [%]	RT ^b^ [h]
Pd-FAU membrane	0.0125 Pd content 1.4% (1.75 mg)	0.026	HCOONa [1]	60	93.90	0.75	0.96	24
Pd–CS–SiO_2_ [53]	0.1 Pd content 3.2%(3.2 mg)	3 × 10^−3^	HCOONH_4_, [8 × 10−3] (in ethanol)	80	-	-	1.2	24

^a^ Hydrogen donor; ^b^ Reaction time.
